# Chrysophanic Acid in Skin Diseases

**Published:** 1879-11

**Authors:** 


### CHRYSOPHANIC ACID IN SKIN DISEASES.

DR. I. NEUMANN records his further experience of this remedy in skin affections. He finds it useful not only in psoriasis and parasitic diseases (such as pityriasis versicolor, herpes tonsurans, eczema marginatum), but also in chloasma uterinum; treated with chrysophanic acid, the latter disfiguring affection may be caused to disappear in a very short time. It exercises a favorable influence also on syphilitic skin diseases and lupus, and its effectiveness seems to be considerably increased by the addition of thymol.—*Glasgow Medical Journal.*

